# Perceived social support and college student engagement: moderating effects of a grateful disposition on the satisfaction of basic psychological needs as a mediator

**DOI:** 10.1186/s40359-022-01015-z

**Published:** 2022-12-11

**Authors:** Zhongyi Xin

**Affiliations:** Faculty of Education, Shaanxi Xue Qian Normal University, No. 101 Shenhe 2nd Road, Changan District, Xi’an, 710100 China

**Keywords:** Perceived social support, Engagement, Grateful disposition, Satisfaction basic psychological needs

## Abstract

**Background:**

Previous research has examined the role of support provided by the workplace in promoting employee engagement. This study aimed to extend this research to the academic environment by testing a proposed model of the relationship between perceived social support and student engagement and its underlying mechanisms, with the latter involving the satisfaction of basic psychological needs and a grateful disposition.

**Methods:**

A total of 622 Chinese college students were selected by convenience sampling. I adopted the Perceived Social Support Scale, Basic Needs Satisfaction in General Scale, Gratitude Questionnaire, and Utrecht Work Engagement Scale-Student to collect their responses. The data were analyzed by using a moderated mediation model with SPSS and the Process 4.0 macro.

**Results:**

The results showed that the satisfaction of basic psychological needs played a mediating role in the association between perceived social support and student engagement, while a grateful disposition played a moderating role. The moderating mediation model further revealed that this effect was more robust for students with a higher grateful disposition than for those with a lower level.

**Conclusion:**

Perceived social support can significantly and positively predict student engagement through the satisfaction of their basic psychological needs. Students with a high grateful disposition benefit more than those with a low grateful disposition from using social support, as well as can use the received social support fully in order to meet their psychological needs and promote academic engagement.

## Introduction

Numerous studies focus on the role of social support in buffering stress [1] and improving mental health [2]. The *thriving through relationships* model suggests that researchers should pay more attention to the role of social support in promoting psychological growth and development [3]. Furthermore, many studies have confirmed the association between organizational support and work engagement [4]. However, insufficient attention has been paid to college students’ engagement with school (student engagement hereafter) and its relationship with their perceived social support. As academic engagement is crucial for school success [5], further research on this matter is needed.

According to self-determination theory and basic psychological needs theory, the interaction between the individual and environment is an essential factor in individuals’ development, especially given that when the external environment meets their basic psychological needs, their well-being and growth can improve [6]. Thus, perceived social support and the satisfaction of basic psychological needs may influence student engagement. However, the relationships among perceived social support, the satisfaction of basic psychological needs, and student engagement remain unclear, as do the underlying mechanisms. In addition, a grateful disposition, a personality trait emphasized in Chinese culture [7], influences the interpretation and use of support received from others [8]. Specifically, higher levels of gratitude may lead to a more compassionate view of the support received from others and a better use of resources to meet one’s inner psychological needs. Therefore, whether different grateful disposition levels influence the effect path between perceived social support and the satisfaction of basic psychological needs remains unknown.

This study aimed to answer the following questions: (1) Does perceived social support strengthen student engagement? (2) Does perceived social support, as a resource in a social situation, improve student engagement by satisfying basic psychological needs? (3) If the relationship between perceived social support and student engagement is mediated by the satisfaction of basic psychological needs, does a grateful disposition have a robust effect on the perceived social support–satisfaction of basic psychological needs relationship? In other words, does a grateful disposition play a moderating role in the first effect path?

### Relationship between perceived social support and student engagement

*Student engagement* is the extent of a student’s cognitive, emotional, and behavioral involvement in academic endeavors [9] and is a critical factor in school success [10]. Therefore, a clear understanding of the factors influencing student engagement is necessary to promote students’ academic success. In previous research, student engagement has been viewed as a self-regulated process [11]. Therefore, studies have focused on the separate effects of the various internal factors influencing student engagement. However, social support, defined as the perception or experience of others’ care and esteem [12], may influence student engagement [13]. According to the *buffer effect of social support* [14], social support acts as a buffer against the stress caused by adversity.

However, managing stress and maintaining a mentally healthy state is the explicit goal of only a minority, with most students striving to attain all-around better development, such as well-being in life and engagement in their studies. Hence, how do students who are not under stress respond when they perceive that they have access to social support? Two models, namely, the *main effect of social support and thriving through relationships* models [3], suggest that social support facilitates a general sense of well-being in life and promotes success in work and study. Hence, it is necessary to focus on the thriving effect of social support. Furthermore, Numerous studies have confirmed the role of social support in promoting well-being [15]; this implies that perceived social support may also strengthen student engagement.

### Mediating role of the satisfaction of basic psychological needs

Self-determination theory is a macro theory of human motivation and personality [16]. It assumes that people are active organisms with a tendency to self-integration and self-improvement. However, this tendency does not occur naturally; it is achieved through the support of the external environment. Basic psychological needs theory, which is a sub-theory of self-determination theory, states that individuals have three basic psychological needs: autonomy, competence, and relatedness [17]. The need for autonomy refers to the sense of control and psychological freedom that an individual feels over their own behavior. Competency needs refer to an individual’s belief that one’s learning behaviors or actions can reach a certain level and that these are competent for the activity. Relatedness needs refer to what an individual feels is necessary for developing good relationships and accessing the others’ support [18]. These three basic psychological needs are important resources for individual adaptation and even prosperity [19]. When they are satisfied, the internal motivation of individuals is stimulated and they show stronger initiative, enthusiasm, and persistence at work and in their study [20, 21].

Research in the field of work has found that the satisfaction of basic psychological needs is significantly positively correlated with work engagement [22], and that students and employees share common characteristics [23]. Therefore, it is reasonable to assume that the satisfaction of students’ basic psychological needs can also positively predict their learning engagement. Abundant studies have confirmed the positive association between the satisfaction of autonomy needs [24], competency needs [25], relatedness needs [26], and engagement. To sum up, it can be inferred that the satisfaction of students’ basic psychological needs can positively predict their learning engagement.

As the *job-demand model* suggests [27], when individuals perceive organizational support, they view it as a resource for satisfying their needs to achieve better development. If their basic psychological needs are not met by the organization, individuals feel frustrated and experience burnout. The theory of basic psychological needs states that autonomy, competence, and relatedness needs are met through the social environment. When individuals are supported by the external environment, they regard this support as a resource to meet their needs. Studies have found that when teachers [28] and families [29] support students’ learning and development, students have more resources to challenge higher goals, allowing them to meet their competence needs. Social support, especially perceived social support, does not interfere with the freedom and autonomy of students, thus also meeting their autonomy needs. Relationship motivation theory states that the satisfaction of competency and autonomy needs can satisfy relatedness needs [30]. Therefore, the higher perceived social support, the higher is the satisfaction of students’ basic psychological needs (autonomous needs, competency needs, relatedness needs), and this satisfaction can then promote student engagement. Therefore, this study hypothesizes that the satisfaction of basic psychological needs plays a mediating role between perceived social support and student engagement.

### Moderating role of a grateful disposition

Various studies have confirmed the contribution of social support to health and well-being. However, some researchers have found that the effects of social support may be ambiguous and even lead to adverse consequences [31]. Further analysis reveals that the quantity, type, and pattern of social support provided may limit its benefits [32]. Nonetheless, few studies consider its effects on the recipient’s personality.

Gratitude has been taken as state gratitude or trait gratitude in previous research. The former, as an affective state, refers to the emotional experience of receiving kindness from others, while the latter is regarded as a personality trait [33], defined as the individual differences in the frequency and intensity of gratitude and tendency to respond to events with a grateful emotion [34]. A grateful disposition is a central moral trait in the Chinese culture [35]. Its importance is emphasized in many social contexts such as school, family, and community. When college students perceive or receive social support from institutions, school staff, and family members, they can in turn increase their academic engagement and performance, and this response is often expected by parents and teachers. However, not everyone receiving support from others can use such resources fully, indicating that different individuals perceive or use social support differently.

Further, levels of gratitude vary among individuals. The social-cognitive model of gratitude states that the level of gratitude affects the perception and understanding of the environment [8], and researchers have shown that the interpretation of the giver’s motivation may diminish or enhance the benefits of support [36]. Individuals with high disposition toward gratitude are more likely to feel grateful and have a more open, kind, and positive attitude toward social support [37]. Coping theory [38] further holds that individuals with a high grateful disposition are better at using resources to meet their psychological needs. By contrast, individuals with low gratitude traits tend to take social support for granted, despise the contributions of others, and even distort the goodwill of social support, resulting in defense and rejection (Lin 2016). Therefore, they do not make full use of social support.

Kashdan et al. found that the key factor behind gratitude is the awareness of various good deeds, which can raise people’s self-confidence and sense of autonomy [39]. Broadens and builds theory [40] holds that gratitude can help individuals develop better close friendships (satisfying relatedness needs) and makes it is easier for people to see support from others as caring, loving, respectful, and delivered in good faith rather than because of interference; hence, people high in gratitude can use support from others fully, thereby satisfying their autonomy and competence needs. Relationship motivation theory [30] states that only after autonomy and competence needs have been met can true intimacy be established, thereby satisfying relatedness needs. Meanwhile, individuals with high gratitude traits seem to be better at using social support to meet their basic psychological needs than are those with low gratitude traits. In conclusion, this study hypothesizes that a grateful disposition plays an important moderating role on the relation between perceived social support and the satisfaction of basic psychological needs.

### Present study

**Fig. 1:**
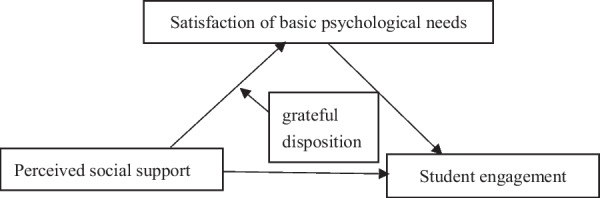
Proposed moderated meditation model

Previous research has emphasized the protective role of social support in individuals’ ability to cope with stress. However, few studies have focused on the role of perceived social support in strengthening student engagement. Further, many studies examine the relationship between organizational support and workplace engagement. Moreover, few researchers have investigated the association between social support and student engagement. Thus, this study examines the association between perceived social support and student engagement based on basic psychological needs theory and the thriving through relationships model. It is proposed that perceived social support promotes student engagement directly and facilitates student engagement by increasing the satisfaction of basic psychological needs indirectly. In addition, a grateful disposition strengthens the association between perceived social support and the satisfaction of basic psychological needs. Figure 1 illustrates the proposed mediated moderation model.

## Methods

### Participants

Seven hundred college students attending universities in Shaanxi and Shanxi Provinces completed the survey. The students were recruited using convenience sampling. Seventy-eight participants were excluded because they provided random responses, leaving 622 valid responses (305 men and 317 women, age range 18–23). The sample included 161 first-, 183s-, 154 third-, and 124 fourth-year students. Ethical approval was obtained from the ethics committee at the researcher’s university.

### Procedure

Twenty school administrators and counselors were recruited to serve as investigators. The researcher trained the investigators before beginning the study. The survey was conducted during the weekly scheduled class meeting. Students receiving professional mental health services were excluded from the survey. This anonymous questionnaire took 10–15 min to complete and was collected immediately and returned to the researcher. The investigation was completed within two weeks, after which the researcher collected the questionnaires and performed data entry and analysis.

## Measures

### Perceived social support

This was the first measure administered. It adopted the subscale of perceived social support from the Two-Way Social Support Scale [41], which measures participants’ perceived social support in recent weeks (e.g., “There is at least one person that I can share most things with”). Six items were rated on a 5-point Likert scale (1 = strongly disagree, 5 = strongly agree), and a higher score indicated more perceived social support. The Cronbach’s α (internal reliability) of the subscale was 0.874.

### Satisfaction of basic psychological needs

Next, the satisfaction of basic psychological needs was measured using the Basic Needs Satisfaction in General Scale [42]. This 21-item scale includes the satisfaction of autonomy needs (e.g., “I feel like I am free to decide for myself how to live my life”), competence needs (e.g., “People I know tell me I am good at what I do”), and relatedness needs (e.g., “I really like the people I interact with”). The participants rated the items on a 5-point Likert scale (1 = not very true of me, 5 = very true of me) with reference to experiences over the past week, and higher scores on the full scale or subscales indicated better levels of needs satisfaction. The Cronbach’s α of the scale was 0.830.

### Grateful disposition

The Gratitude Questionnaire [34] was used to measure the tendency to feel grateful. Its six items (e.g., “I am grateful to a wide variety of people”) were rated on a 5-point Likert scale (1 = strongly disagree, 5 = strongly agree), and a higher score indicated that the individual is more likely to experience feelings of gratitude. The Cronbach’s α of the scale was 0.805.

### Student engagement

Finally, the Utrecht Work Engagement Scale-Student was used to assess student engagement [43]. It comprises 17 items covering three dimensions: vigor (e.g., “When studying, I feel strong and vigorous”), dedication (e.g., “My studies inspire me”), and absorption (e.g., “Time flies when I’m studying.”). Items are responded on a 5-point Likert scale (1 = not very true of me, 5 = very true of me), and a higher subscale score indicated stronger student engagement. The Cronbach’s α was 0.914 for the full scale and 0.803, 0.887, and 0.800 for the vigor, dedication, and absorption subscales, respectively.

## Results

### Common method bias test

Harman’s single-factor test evaluated the degree of common method bias before the preliminary analyses. The results showed eight factors with eigenvalues above 1. The first factor explained 23.66% of the variance, which was below the recommended value of 40% [44]. The model-fitting indices (*χ*^*2*^/*df* = 6.218, *CFI* = 0.472, *TLI* = 0.454, *RMSEA* = 0.092, *SRMR* = 0.109) did not meet the requirements according to the confirmatory factor analysis of the single-factor model with *R* using the “*lavaan” package*. Therefore, no common method bias was detected in this study.

### Preliminary analyses

Table 1 presents the means (*M*), standard deviations (*SD*), and correlation coefficients of the variables. Perceived social support was associated with student engagement (*r* = 0.391, *p* < 0.01), the satisfaction of basic psychological needs (*r* = 0.532, *p* < 0.01), and a grateful disposition (*r* = 0.254, *p* < 0.01). Further, the satisfaction of basic psychological needs was associated with student engagement (*r* = 0.425, *p* < 0.01) and a grateful disposition (*r* = 0.326, *p* < 0.01), while a grateful disposition was associated with student engagement (*r* = 0.321, *p* < 0.01).

**Table 1:** Descriptive statistics and correlations of the variables

	M ± SD	Gender	Age	PSS	SBPN	Engagement	GD
Gender	1.51 ± 0.5	1					
Age	19.79 ± 1.609	0.513**	1				
PSS	3.711 ± 0.748	− 0.262**	− 0.201**	1			
SBPN	3.315 ± 0.413	− 0.290**	− 0.137**	0.532**	1		
Engagement	3.477 ± 0.569	− 0.449**	− 0.320**	0.391**	0.425**	1	
GD	3.714 ± 0.564	− 0.252**	− 0.178**	0.254**	0.326**	0.321**	1

PSS, perceived social support; GD, a grateful disposition; SBPN, satisfaction of basic psychological needs

**p < 0.01

### Mediation analyses

**Fig. 2:**
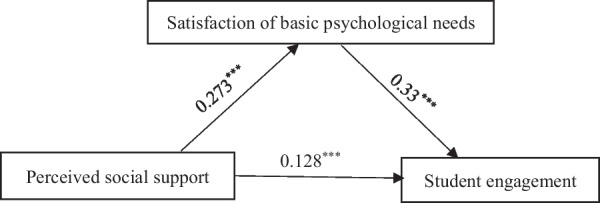
Mediating effect of the satisfaction of basic psychological needs

Model 4 of the Hayes SPSS Process 4.0 Macro [45] (Hayes 2013) was used to test the mediating effect of the satisfaction of basic psychological needs in the relationship between perceived social support and student engagement. Model 4 is the basic mediation model, which consists of predictor variable X (perceived social support in this study), mediating variable M (satisfaction of basic psychological needs), and outcome variable Y (student engagement). If X can significantly predict M (path coefficient a), M can significantly predict Y (path coefficient b), and a*b is significantly different from zero, then the mediating effect is valid. Figure 2 and Table 2 show that perceived social support was associated with a higher satisfaction of basic psychological needs, *b* = 0.273, *t* = 14.235, *p* < 0.001, 95% CI [0.235, 0.311], and that a higher satisfaction of basic psychological needs was associated with increased student engagement, *b* = 0.33, *t* = 6.027, *p* < 0.001, 95% CI [0.223, 0.438]. Moreover, perceived social support was associated with increased student engagement, *b* = 0.128, *t* = 4.248, *p* < 0.001, 95% CI [0.069, 0.187]. The indirect effect was 0.09, 95% CI [0.055, 0.128], and the full standardized indirect effect was 0.118, 95% CI [0. 073, 0.168]. Thus, the satisfaction of basic psychological needs plays a mediating role on the association between perceived social support and student engagement.

**Table 2:** Mediating effect of the satisfaction of basic psychological needs

Predictor	Model 1: basic psychological needs				Model 2: student engagement			
	*b*	*t*	95% CI		*b*	*t*	95% CI	
Gender	− 0.158	− 4.839	− 0.223	− 0.094	− 0.317	− 6.986	− 0.406	− 0.228
Age	0.016	1.559	− 0.004	0.035	− 0.039	− 2.853	− 0.066	− 0.012
PSS	0.273	14.235***	0.235	0.311	0.128	4.248***	0.069	0.187
SBPN					0.33	6.027***	0.223	0.438
*R2*	0.311				0.328			
*F*	92.746***				75.182***			

*N* = 622

PSS, perceived social support; SBPN, satisfaction of basic psychological needs

***p < 0.001

### Moderated mediation analyses

Model 7 of the Hayes SPSS Process 4.0 Macro [45] was used to test the moderated mediation model. Model 7 is based on Model 4, but adds the conditional variable M_O_ (a grateful disposition in this study). If M_O_ moderates the relationship between X and M and the coefficient of X*M_O_ is significantly different from zero, the moderating effect of M_O_ is established. Table 3 shows that the interaction of perceived social support and a grateful disposition was associated with the increased satisfaction of basic psychological needs, *b* = 0.087, *t* = 2.986, *p* < 0.01, 95% CI [0.03, 0.145].

**Table 3:** Moderated mediation effect of perceived social support on student engagement

Predictor	Model 1: satisfaction basic psychological needs				Model 2: student engagement			
	*b*	*t*	95% CI		*b*	*t*	95% CI	
Gender	− 0.128	− 3.983	− 0.192	− 0.065	− 0.317	− 6.986	− 0.406	− 0.228
Age	0.015	1.534	− 0.042	0.034	− 0.039	− 2.853	− 0.066	− 0.012
PSS	0.252	13.231***	0.214	0.289	0.128	4.248***	0.069	0.187
GD	0.134	5.321***	0.084	0.183				
PSS × GD	0.087	2.986**	0.03	0.145				
SBPN					0.33	6.027***	0.223	0.438
*R2*	0.35				0.328			
*F*	66.183***				75.182***			

*N* = 622

PSS, perceived social support; GD, a grateful disposition; SBPN, satisfaction of basic psychological needs

***p < 0.001, **p < 0.01

**Fig. 3:**
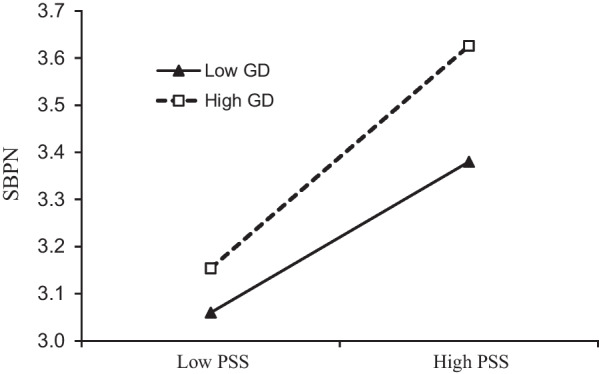
Moderating effect of a grateful disposition in the relationship between perceived social support and the satisfaction of basic psychological needs. *Note:* PSS, perceived social support, GD, a grateful disposition; SBPN, satisfaction of basic psychological needs

The mean of a grateful disposition plus (minus) one standard deviation was defined as a high (low) grateful disposition. Figure 3 shows that the positive relationship between perceived social support and the satisfaction of basic psychological needs was stronger when the level of a grateful disposition was high, *b* = 0.301, *t* = 12.081, *p* < 0.001, 95% CI [0.252, 0.35], than when it was low, *b* = 0.203, *t* = 7.597, *p* < 0.001, 95% CI [0.153, 0.253]. The moderated mediation index was 0.029, 95% CI [0.006, 0.060]. When the level of a grateful disposition was high, the moderated indirect effect, 0.099, 95% CI [0.057, 0.146], was stronger than when it was low, 0.067, 95% CI [0.04, 0.096].

## Discussion

The present study examined the roles of the satisfaction of basic psychological needs and a grateful disposition in the association between perceived social support and student engagement. It found a positive correlation between perceived social support and student engagement. In addition, a mediating effect of the satisfaction of basic psychological needs and a moderating effect of a grateful disposition were observed in this relationship.

### Mediating role of the satisfaction of basic psychological needs

Previous research has found a positive relationship between organizational support, a form of social support, and work engagement. This study extends the effect of social support from work engagement to student engagement. Similarly, the results confirm that perceived social support is associated with increased student engagement. The effect (r = 0.39) of perceived social support on student engagement was similar to that for employees in the workplace in a prior study [46] (r = 0.32; Halbesleben 2010). The research findings align with the model of thriving through relationships [3], a framework that proposes that social support protects individuals from stress in adversity and facilitates personal growth and development.

This study identified the mechanisms by which perceived social support influences student engagement, namely, through the satisfaction of basic psychological needs. Thus, the relationships shown in prior research between perceived social support and the satisfaction of basic psychological needs [47] as well as between the satisfaction of basic psychological needs and student engagement [48] were confirmed. This study focused on the relationships among perceived social support, the satisfaction of basic psychological needs, and student engagement. The results were consistent with those of previous studies, verifying claims founded on basic psychological needs theory that support and resources that help individuals feel more autonomy, competence, and relatedness also help students engage more in school and related activities [49] The results of this study suggest that schools and families should create more supportive resources to promote students’ engagement, especially the social support may be capable of satisfying students’ basic psychological needs for autonomy, competence, and relatedness.

### Moderating role of a grateful disposition

This study found that a grateful disposition played a moderating role in the relationship between perceived social support and the satisfaction of basic psychological needs. Previous studies have indicated that when social support is inappropriately provided (e.g., excessively or incongruently in relation to own needs), the receiver is inclined to focus on a sense of incompetence and on the support provider’s negative evaluation [31]. Providing “invisible” support is one way to alleviate the pressure of receiving social support [50]. This study concentrated on the strengthen effect of a grateful disposition. Students with higher levels of gratitude appraise the support received from a social situation more positively. They also more readily regard support as valuable, costly to provide, and more altruistically intended, whether received or perceived. Thus, students with such traits focus on the full use of the support and how to repay the provider, rather than on own feelings of incompetence and embarrassment, allowing them to benefit more from social support than individuals with a low grateful disposition.

The results of this study, thus, suggest that an intervention for individuals with a low grateful disposition to improve their tendency to be grateful may promote their goodwill understanding of social support, as well as help them use social resources fully to satisfy their psychological needs.

### Limitations and implications

Several limitations should be considered when interpreting the study’s findings. First, because a cross-sectional design was used, the moderated mediation path model cannot be assumed to be effective. Future research should employ longitudinal and experimental designs to find more robust evidence and build a causal model.

Second, social desirability bias and random answering may have limited the validity of the data gathered using self-report methods. A more reliable strategy would be to collect data from students and bystanders such as teachers and parents simultaneously.

Third, both perceived social support and the satisfaction of basic psychological needs were measured with a general focus. Because of this, practical implications cannot be formulated on which specific source (e.g., family, peers, and teachers) of perceived social support increases student engagement.

Finally, the study samples were selected from four universities in northwestern China. A representative sample from different regions and school sections may produce more generalizable findings.

Despite the limitations mentioned above, the results confirmed the association and underlying mechanisms between perceived social support and student engagement. In addition, it confirmed the role of perceived social support in promoting personal growth and extended the study of engagement from employees in the workplace to students in college. The findings suggest that increasing perceived social support may be a potential path to improving academic engagement, especially if the provided support is able to meet the basic psychological needs of students. In addition, interventions targeting individuals with a low grateful disposition may prove useful in strengthening the relationship between perceived social support and academic engagement among these students.

## Conclusion

The study’s results indicate that perceived social support strengthens student engagement. Furthermore, the mediation analysis reveals that the satisfaction of basic psychological needs is one possible mechanism underlying this association. The moderated mediation analysis reveals that a grateful disposition strengthens the relationship between perceived social support and the satisfaction of basic psychological needs. The connection between perceived social support and the satisfaction of basic psychological needs is stronger for students with high levels of grateful disposition than those with lower levels.

## Data Availability

The datasets used and analyzed during the study are available from the corresponding author on reasonable request.
